# Neonatal exposure to permethrin pesticide causes lifelong fear and spatial learning deficits and alters hippocampal morphology of synapses

**DOI:** 10.1186/1866-1955-6-7

**Published:** 2014-03-29

**Authors:** Cinzia Nasuti, Patrizia Fattoretti, Manuel Carloni, Donatella Fedeli, Massimo Ubaldi, Roberto Ciccocioppo, Rosita Gabbianelli

**Affiliations:** 1 School of Pharmacy, Pharmacology Unit, University of Camerino, Via Madonna delle Carceri, Camerino, MC 62032, Italy; 2 Cellular Bioenergetics Laboratory, INRCA Scientific Technological Area, Via Birarelli 8, Ancona 60121, Italy; 3 School of Pharmacy, Molecular Biology Unit, University of Camerino, Via Gentile III da Varano, Camerino, MC 62032, Italy

**Keywords:** Neonatal exposure, permethrin, hippocampus, synapse, fear conditioning

## Abstract

**Background:**

During the neurodevelopmental period, the brain is potentially more susceptible to environmental exposure to pollutants. The aim was to determine if neonatal exposure to permethrin (PERM) pesticide, at a low dosage that does not produce signs of obvious abnormalities, could represent a risk for the onset of diseases later in the life.

**Methods:**

Neonatal rats (from postnatal day 6 to 21) were treated daily by gavage with a dose of PERM (34 mg/kg) close to the no-observed-adverse-effect level (NOAEL), and hippocampal morphology and function of synapses were investigated in adulthood. Fear conditioning, passive avoidance and Morris water maze tests were used to assess cognitive skills in rats, whereas electron microscopy analysis was used to investigate hippocampal morphological changes that occurred in adults.

**Results:**

In both contextual and tone fear conditioning tests, PERM-treated rats showed a decreased freezing. In the passive avoidance test, the consolidation of the inhibitory avoidance was time-limited: the memory was not impaired for the first 24 h, whereas the information was not retained 72 h following training. The same trend was observed in the spatial reference memories acquired by Morris water maze. In PERM-treated rats, electron microscopy analysis revealed a decrease of synapses and surface densities in the stratum moleculare of CA1, in the inner molecular layer of the dentate gyrus and in the mossy fibers of the hippocampal areas together with a decrease of perforated synapses in the stratum moleculare of CA1 and in the inner molecular layer of the dentate gyrus.

**Conclusions:**

Early-life permethrin exposure imparts long-lasting consequences on the hippocampus such as impairment of long-term memory storage and synaptic morphology.

## Background

Pyrethroids are among the most frequently used pesticides. The primary source of exposure is believed to be through diet. Additional exposure via ingestion of contaminated household dust may occur after indoor application of pesticides such as permethrin (PERM), the main pyrethroid used for indoor pest control, in pet shampoos and treatment of wood furniture.

It has been suggested that this class of pesticides shares the same mode of action: they modify the voltage-gated sodium channels (VGSCs) such that they remain open for longer periods of time than under normal conditions, leading to neurotoxicity [1].

The presence of pyrethroid metabolites, such as 3-phenoxybenzoic acid, in the urine of U.S. and German residents indicates that the population is widely exposed to this family of pesticides; furthermore, children have higher exposures than adolescents and adults [2-5].

Brain susceptibility to pollutants is modulated according to the period of the human lifespan in which exposure occurs; for example, developing brain is potentially more susceptible to the toxic effects of pesticides compared to the adult brain due to lower metabolic detoxification. Previous experiments in our lab showed that pup rats are 4 to 17 times more vulnerable to the acute toxicity of pyrethroids than adult rats due to their lower capacity for metabolic detoxification [6]. However, other factors might contribute to or work in concert with an immature metabolic capacity of developing animals to increase their susceptibility to pyrethroids. Meacham and coworkers [7] demonstrated that the NaV 1.3 isoform of the mammalian sodium channel, which is highly expressed during development in rodents, is more sensitive to some pyrethroids.

The U.S. Environmental Protection Agency (EPA) has determined a reference dose (such as an estimate of the quantity of chemical that a person could be exposed to every day of its life with no appreciable risk of adverse health effects) to be 0.25 mg/kg/day for both acute and chronic dietary exposures to permethrin [8]. This level is based on a no-observed-adverse-effect level (NOAEL) of 25 mg/kg/day in rats. However, recent publications have suggested that there may be significant aspects that were not considered in the original evaluation of pyrethroid toxicity. For example in neurodevelopmental studies, repeated exposure to pyrethroids at dosage close to the NOAEL that do not produce frank poisoning or signs of obvious abnormalities (for example, body weight loss, red eyes, *etcetera*) in neonates may cause long lasting effects in adult age [9-15]. In previous studies, we exposed neonatal rats to PERM during the developmental period until weaning at 21 days, at a dose of 34 mg/kg, close to the NOAEL. In adolescent age, the animals, aged 35 days, previously treated with the insecticide showed lower dopamine levels in the striatum together with a glutathione depletion [10]. In pre-senescent age, alterations in working memory processes were the long lasting effects observed in these rats, whereas no disturbance in the acquisition phase of a hidden-platform Morris water maze (MWM) was observed across 5 days of training. These behavioral effects were associated with a decrease in dopamine and an increase in noradrenaline in the hippocampus of PERM-treated rats as compared to controls at pre-senescent age [11].

Hence, in the present study we sought to explore whether early life exposure to PERM would have resulted also in long lasting alteration of hippocampal morphology and function as measured in memory consolidation tasks in adulthood. The rats were treated, as in previous studies, at a dose of 34 mg/kg from postnatal day (PND) 6 to PND 21. This period represents a period of active neurodevelopment in the rat [16,17] and has been associated with the development of several diseases in adulthood and aging [18,19]. To test the hypothesis, we conducted three main experiments. The primary goal of the study was to investigate whether previous neonatal exposure to low levels of pesticide could impair the consolidation process of memories in rats later in adult age using fear-motivated tests such as fear conditioning (experiment 1) and passive avoidance (experiment 3). Two variants of fear conditioning, termed contextual and cue fear conditioning [20], have been used as behavioral probes for detecting whether hippocampus and amygdala functions were intact, respectively. Besides, the functionality of the hippocampus was characterized using a hidden-platform Morris water maze task that permitted to assess the consolidation process of spatial memory previously acquired in the training (experiment 2). In order to evaluate if PERM exposure would have also altered the neuroanatomy of the hippocampus, adult rats treated in early life with the pesticide or vehicle were subjected to morphological analysis to measure synaptic numeric density, surface density, average area and the percent of perforated synapses. The stratum moleculare of CA1 (SMCA1), the inner molecular layer of the dentate gyrus (IMLDG) and the mossy fibers (MF) of the hippocampus were investigated. Alteration of the synaptic structural pattern in the hippocampus is correlated with disruption of the memory consolidation process. This implies that the morphology of a spine directly reflects its function; a correlation between reductions of spine densities in the hippocampus and declines of hippocampal memory has been demonstrated in aging and pathological conditions [21,22].

## Methods

### Materials

Technical grade (75:25, trans:cis; 94% purity) 3-phenoxybenzyl-(1R,S)-cis,trans-3-(2,2-dichlorovinyl)-2,2-dimethylcyclopropanecarboxyl-ate, PERM (NRDC 143) were generously donated by Dr. A. Stefanini of ACTIVA (Milan, Italy). Corn oil, glutaraldehyde, ethanol, phosphotungstic acid and a Durcupan resin kit were obtained from Sigma (Milan, Italy).

### Animals

Behavioral and morphological experiments were performed on six-month old male Wistar rats (weight, 600 to 700 g) born in our laboratory from primiparous dams, in accordance with the guidelines laid down by the European Communities Council (86/609/ECC) for the care and use of laboratory animals. The study was approved by the Ethical Committee of the University of Camerino. The animals were housed in plastic (Makrolon) cages (two rats per cage) in a temperature-controlled room (21 ± 5°C) and 60% humidity on 12 h light/dark inverted cycle (light was switched on at 7:00 p.m.) and maintained on laboratory diet (Mucedula, Italy) with water *ad libitum*.

The parturition day was set as PND zero. On PND1, all litters were examined externally for the presence of gross abnormalities, sexed, weighed and the female pups were discarded. At 6 days of age, litters (n = 32) were randomly assigned to two experimental groups (control and PERM treated). At PND21, two male pups of each litter were assigned to one of two experimental groups, whereas the others were used for other studies. For the experiments, the groups of animals were formed by drawing them from different litters so that no group contained siblings. All experiments were conducted blind to the treatment.

### Treatment

In this study, we tested only one dose of PERM that is close to the NOAEL so that it would not have adverse health effects on the rats.

PERM was dissolved in corn oil and administered by gavage at a dose of 4 ml/kg, that is, 1/10 of LD_50_ calculated at ages P8 to P21. The LD_50_ determined in young rats 8 to 21 days old was 340 to 471 mg/kg according to Cantalamessa [6]; thus 34.05 mg/kg was the dose of PERM.

The compounds were prepared fresh daily and administered once a day from PND6 to PND21. Rats from control group were treated with vehicle (corn oil, 4 ml/kg) on a similar schedule. In this study, we did not include a control group not fed with corn oil since no behavioral and neurochemical differences were observed between the control group not treated with corn oil and the control group treated with vehicle in our previous works (unpublished data, C.N).

The volume of the compound administered was adjusted daily on the basis of the measured body weight. On PND21, the offspring were weaned and the littermates were housed together. Rats were weighed at 1-week intervals throughout the course of study.

### Procedure

At 6 months of age (adult), a first batch of animals (13 control and 15 PERM-treated rats) was subjected to fear conditioning (PND180 to PND183), open field (OF) (PND184), elevated plus maze (EPM) (PND185), nociceptive tests (PND186 to PND187) and then all animals were sacrificed (PND188) and the hippocampi were processed for morphological analysis of synapses. A second batch of animals (18 controls and 18 PERM-treated rats) was subjected in succession to MWM (PND180 to PND188) and passive avoidance test (PND189 to PND192). All experiments were performed during the animal’s dark cycle with testing performed from 8:00 to 13:00 a.m.

### Experiment 1. To test adult rats, exposed neonatally to permethrin, in fear conditioning paradigms

To evaluate whether the neonatal treatment with PERM induced deficits in fear-related memory in adulthood, both control (n = 13) and PERM-treated (n = 15) rats were tested for context and cued fear conditioning according to the partially modified procedure of Goosens and Maren [20].

Briefly, behavioral testing was conducted in three sessions. In the first session, or conditioning day (PND180), rats were allowed to explore the context for 3 min prior to tone-shock conditioning in chambers (MED Associates) equipped with a video camera that was connected to EthoVision 7.0 software (Noldus Information Technology, Wageningen, The Netherlands). The latter was used to control shock periods/amplitude, cue presentation (for cued fear conditioning), and to measure experimental parameters such as time trial, freezing, and locomotor activity, which was defined as distance moved. After the 3-min habituation period, a 10-s tone (9 kHz, 80 dB) was presented that co-terminated with a 2-s electric foot shock (1.0 mA). The rats were exposed to 10 conditioned-unconditioned stimuli (CS-US) pairings with a 60-second stimulus-free period or intertrial interval (ITI) between pairings. During the ITI, the rats were scored for percent time freezing as a measure of fear. In the second session, or context extinction test (PND181), rats were returned to the chamber (in absence of the tone and foot shock) and the percent freezing was scored for 8 min. In the third session, or tone extinction test (PND182), fear conditioning to the conditioned stimulus (CS) was tested in a new context that was created by inserting a black plastic bucket (different size, form and floor) into the test chamber. In the new context, the chamber was cleaned with a different cleaner than that used during the conditioning and contextual testing to provide a specific odor. Following a 3-min habituation period, a tone was presented in the absence of foot shock for 3 min while the last 5 min were performed without audible cue. The animals were scored for percent time freezing during the first 3 min (before the tone) and during the 8 min of tone extinction. EthoVision software was set for the dynamic subtraction method to score understood freezing (immobility threshold: 5%; highly mobile threshold: 95%; mobility averaging interval: 20) as the absence of all movement except respiration. Preliminary experiments were performed to optimize the accuracy between automated and observer scoring in order to validate the output measures of the software at the setting chosen.

### Experiment 2. To test adult rats, exposed neonatally to permethrin, in a Morris water maze task

A second batch of animals (18 control and 18 PERM-treated rats) was subjected to training sessions (PND180 to PND185) in a MWM that consisted of four trials each day and with a hidden platform positioned in the same quadrant of the pool throughout sessions as reported in Nasuti *et al*. [11]. On the last day of training, the animals were divided in two groups: one group (9 control and 9 PERM-treated rats) was submitted to a probe test 24 hours after last training session, whereas the second group (9 control and 9 PERM-treated rats) was given a probe test 72 hours later. In the probe test, the platform was removed from the pool and the rat was allowed to search for it for 90 s; rats that have adopted a spatial strategy will search focally near the former location of the platform. Measurement of time spent in the quadrant in which the escape platform had previously been located permits an assessment of whether there are any differences on long-term spatial reference memory (24 h or 72 h) between control and PERM-treated groups. Behavioral data from the probe tests were acquired and analyzed using an automated tracking system (EthoVision 7.0 software).

### Experiment 3. To test adult rats, exposed neonatally to permethrin, in a passive avoidance task

The same set of animals used for experiment 2 (18 control and 18 PERM-treated rats) was submitted to a training test (PND189) in a passive avoidance task and then divided into two groups (9 control and 9 PERM-treated rats): one group was subjected to a memory retention test 24 hours (PND190) after the training test, whereas the second group was submitted to a retention test 72 hours (PND192) later.

The passive avoidance box (16 × 16 × 18 cm high) consisted of a light compartment connected to a dark compartment by a guillotine door. Electric shock was delivered to the grid floor by a constant current generator (Med Associates). During the training test, the rats were placed in the light chamber and 60 s later the sliding guillotine door was opened. After the rats entered the dark compartment, the door was closed and a foot shock of 1.24 mA (3 s) was given. Then, rats were replaced in the light compartment and if they did not re-enter the dark compartment in 300 s they were removed to the apparatus. If the rats re-entered the dark compartment before fulfillment of the avoidance criteria of 300 s, the shock treatment was administered again. In the training test, the latency of entrance into the dark chamber was recorded and rats with the latency greater than 300 s were excluded from the study. In the retention test, passive defensive reactions, assessed in terms of the latent period of transfer from the light to the dark compartment, were tested 24 or 72 h after foot shock. For this, the rat was replaced in the training context (light compartment) and the latency to enter the dark compartment was measured up to a maximum of 300 s as cut-off.

### Experiment 4. To test adult rats, exposed neonatally to permethrin, in open field and elevated plus maze tasks

From the same set of animals used for Pavlovian fear conditioning, 10 controls and 10 PERM-treated rats were subjected to the OF test for 10 min, at PND184 and the EPM for 5 min at PND185, in order to determine the locomotor activity and the anxiety-like behavior, respectively. Briefly, procedures and parameters measured in both tests were reported in [11].

### Experiment 5. To test pain sensitivity of adult rats, exposed neonatally to permethrin

The same batch of animals described above (13 control and 15 PERM-treated rats) was tested for pain sensitivity in a hot plate test at PND186 and in a tail immersion test at PND187 to determine whether there were any differences between control and PERM-treated groups.

Pain reflexes in response to a thermal stimulus were measured using a hot plate apparatus (25.4 × 25.4 cm) (Ugo Basile, Italy), which was surrounded by an opened-top acrylic cage (19 cm tall) [23]. The heated surface of a hot plate was maintained at 50 ± 0.5°C and each rat was gently placed on the plate. The time until the animal either licked its hind paw or jumped in an attempt to escape the hot plate was measured (latency to respond). To avoid tissue damage the cut-off time or latency response was taken as 15 s.

In the tail immersion test, the painful reactions in animals were prompted by a thermal stimulus, that is, by dipping the tip of the tail in hot water. Before the test, each animal was adapted to be handled. The lower 5 cm portion of the tail was dipped in a hot water bath maintained at 50 ± 0.5°C [24]. The time in seconds to withdraw the tail clearly out of the water was taken as the reaction time (cut-off time was 10 s).

### Experiment 6. To analyze the synaptic morphology of adult rats, exposed neonatally to permethrin

One month after completion of the nociceptive tests, six control and six PERM-treated rats were sacrificed by CO_2_ and the right hippocampus of each was fixed in 2.5% phosphate-buffered glutaraldehyde (pH 7.4, 4°C) for 24 h to be processed for ultrastructural analysis of the synapses. Then, the hippocampi were sectioned into thin slices and incubated for 1 hour at 60°C in 1% E-PTA according to the ethanol phosphotungstic acid preferential staining procedure as previously described [25]. Samples were then washed, dehydrated, and embedded in Durcupan resin. Since the synaptic staining was very sharp, the ultrathin tissue sections did not need further contrasting procedure; indeed, E-PTA allows easy visualization of synaptic junctions against a faint background and permits reliable identification and quantitative assessment.

Synaptic sampling was carried out in the SMCA1, in the IMLDG and in the MF of hippocampus in accordance with the ‘equal opportunity rule’ criterion [26] with a transmission electron microscope (EM 900, Carl Zeiss, Germany) connected to a computer-assisted image analysis system (Kontron KS 300). One hundred and fifty fields (each of 9.53 μm^2^) per animal were sampled; this yielded a total surface of 1429.50 μm^2^ per animal. The synaptic numeric density (Nv: number of synapses/μm^3^ of tissue), surface density (Sv: overall area of synaptic junctional zones/μm^3^ of tissue), and average area of synapse (S) were calculated by morphometric formulas. The percentage of perforated synapses (PS) was also evaluated.

### Statistical analysis

Results are expressed as mean ± S.E.M. The contextual and cued fear conditioning, the passive avoidance, the MWM, the OF, the EPM, the tail immersion and hot plate tests, and the ultrastructural features of synapses were analyzed by mean of Student's t test. For the conditioning session, a two-way ANOVA with one factor within (time or tone) and one factor between (treatments) was employed and appropriate post-hoc analysis was carried out using the Newman-Keuls test. Statistical significance was set at *P* < 0.05.

## Results

### General observations

No clinical signs ascribed to PERM or any difference in body weight for PERM-treated animals was observed during treatment or at term as reported from previous studies [11].

### Experiment 1. Freezing activity in adult rats exposed neonatally to permethrin

**Figure 1:**
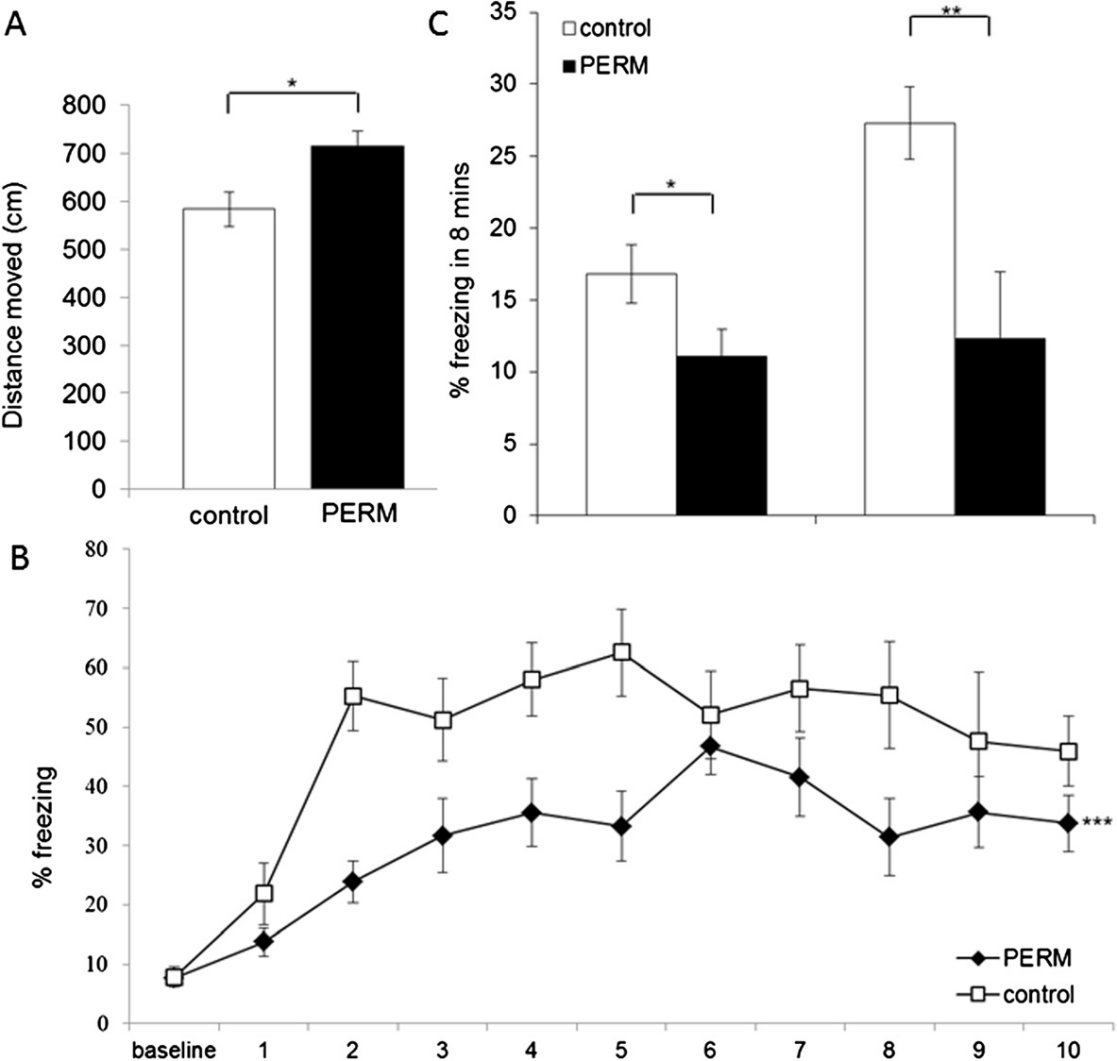
**Fear conditioning tests in PERM-treated and control groups. A)** Locomotor activity, expressed as distance moved, during 3-minute habituation period preceding the conditioning session. **B)** Freezing on conditioning session expressed as 1-minute averages for the period before (baseline) and after each of ten tone-shock conditioning trials. **C)** Freezing on context (CX) and cued (CS) fear conditioning sessions during an 8-minute extinction test. All data are means ± SEM. Group sizes: control (N = 13), PERM (N = 15). *P < 0.05 versus control group; **P < 0.01 versus control group; ***P < 0.001 versus control group.

During the 3-min baseline period prior to tone-shock conditioning, freezing activity was similar between control and PERM-treated groups (data not shown), whereas the locomotor activity, defined as distance moved, was significantly higher in the PERM treated rats (df = 27, t = -2.0762, *P* < 0.05) as shown in Figure 1A.

During the conditioning session, there was an increased freezing in control and PERM-treated rats over the course of tone presentation, indicating successful conditioning to the 10 tones (Figure 1B). However, control and PERM-treated groups did not acquire similar amounts of conditioned freezing to the tone: the percent of freezing in PERM-treated group was lower along the 10 conditioning trials with respect to that of control rats as revealed by a main effect of treatment (*F*(1,27) = 23.05, *P* < 0.001). No significant treatment x tone interaction (*F*(9, 243) = 1.65, *P* > 0.05) was observed.

In both context and cued fear conditioning sessions, significant differences between groups were observed as shown in Figure 1C. Statistical tests showed that the PERM-treated group displayed a significant impairment in context fear conditioning (df = 27, t = -2.069, *P* < 0.05) manifested as reduced freezing response in comparison to the control group during the retrieval testing. In addition, statistical test revealed that the PERM-treated group demonstrated a statistically significant impairment in tone fear conditioning (df = 27, t = -2.970, *P* < 0.01) as shown by decreased freezing when compared to control group during the retrieval testing (8 min). In the pre-tone baseline period of tone fear conditioning, no differences in percent of freezing were observed between control (3.10% ± 0.96) and PERM-treated (2.54% ± 0.56) groups (df = 27, t = -0.510, *P* > 0.05). Therefore, for cue learning, the percent of freezing relative to baseline on that same day were 24.19% and 9.74% for control and PERM-treated groups, respectively.

These data suggested that the PERM-treated rats displayed a significant deficit in freezing behavior after both contextual and tone fear conditioning compared with the control rats.

### Experiment 2. Retention of spatial memory in adult rats exposed neonatally to permethrin

During one week of place training, control and PERM-treated groups did not reveal differences in spatial learning performance (data not shown).

**Figure 2:**
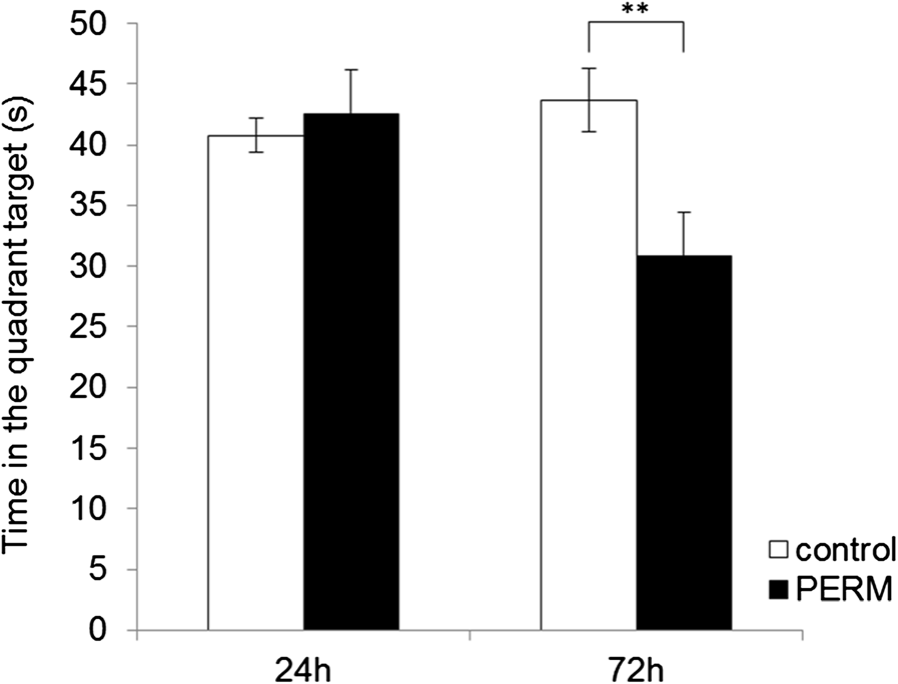
**Performance of PERM-treated and control group on the Morris water maze task in the probe tests at 24 or 72 hours after the last training session.** The time spent in the quadrant target without the platform was measured in 90 seconds. All data are means ± SEM. Group sizes: control (N = 9), PERM (N = 9). **P < 0.01 versus control group.

Figure 2 shows the time spent in the quadrant in which the escape platform had been previously located by both control and PERM-treated groups in the probe tests. Twenty-four hours after training, statistical tests computed on time spent in the target quadrant revealed no significant differences between control and treated rats of a first set (df = 16, t = 0.470, *P* > 0.05). On the contrary, when the probe test was conducted 72 hours after training on a second set of animals, statistical analysis indicated that time spent in the target quadrant was reduced by the treatment with PERM (df = 16, t = -2.941, *P* < 0.01). The latter result demonstrated that the retention of spatial memory was impaired in the PERM-treated rats of the second set because of longer retention intervals (72 h after last training) with respect to the first set submitted to 24-h retention test.

### Experiment 3. Retention of avoidance responding in adult rats exposed neonatally to permethrin

**Figure 3:**
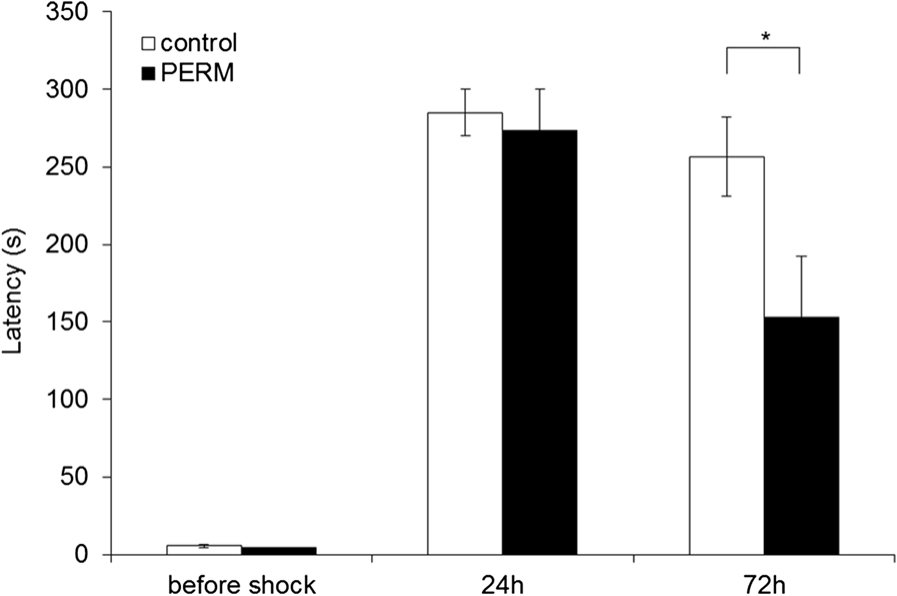
**Performance of permethrin (PERM)-treated and control group on passive avoidance task.** Latency time to enter into the dark sector of the chamber was measured before, 24 hours and 72 hours after shock conditioning. All data are means ± SEM. Group sizes: control (N = 9), PERM (N = 9). *P < 0.05 versus control group.

In the training test, the treatment with PERM in neonatal age did not have effect on the entry latency because both control and PERM treated rats had the same performance (df = 16, t = 1.207, *P* > 0.05) as shown in Figure 3.

Statistical analysis computed on passive avoidance behavior of the first set of animals (df = 16, t = 0.381, *P* > 0.05) showed no significant treatment differences in the test performed 24 h after the training test. On the contrary, entry latencies of second set of animals measured 72 h after the training test were reduced by the treatment with PERM (df = 16, t = 2,190, *P* < 0.05). These data demonstrated that PERM treatment impaired the retention of avoidance responding when more time had elapsed since the day of training.

### Experiment 4. Locomotor activity and anxiety-like responses in adult rats exposed neonatally to permethrin

The results are reported in Table 1. In the OF test, statistical analysis revealed no differences between control and PERM-treated groups in the locomotor activity (ambulatory counts: df = 18, t = -1.300, *P* > 0.05; stereotypic counts: df = 18, t = 1.764, *P* > 0.05; rearing counts: df = 18, t = 1.764, *P* > 0.05). Statistical test also revealed absence of differences in anxiety-like behavior (% ambulatory counts: df = 18, t = 0.105, *P* > 0.05; percent stereotypic counts: df = 18, t = -0.939, *P* > 0.05; percent rearing counts: df = 18, t = -0.854, *P* > 0.05) occurring in the central area of the OF.

**Table 1:** Indices of thermal nociceptive sensitivity (tail-flick latency and hot-plate latency), locomotor activity (number total ambulatory, stereotypic and rearing counts) and anxiety-like behavior (percent ambulatory counts, percent stereotypic and rearing counts in central zone, percent time in open arm and percent open arm entries) measured in control and permethrin (PERM)-treated groups

**Test**	**Parameter**	**Number of animals**	**Control group**	**PERM group**
Tail immersion	Tail flick latency (s)	13 control	4.42 ± 0.32	5.47 ± 0.41
		15 PERM		
Hot-plate	Hot-plate latency (s)	13 control	2.73 ± 0.32	2.43 ± 0.17
		15 PERM		
Open field	Number of total ambulatory counts	10 control	3,374.80 ± 193.13	3,039.40 ± 170.89
		10 PERM		
	Number of total stereotypic counts		1,350.89 ± 156.67	1,714.60 ± 133.95
	Number total rearing counts		167.80 ± 13.62	198.30 ± 10.65
	Percent ambulatory counts in central zone		4.54 ± 0.68	5.04 ± 0.32
	Percent stereotypic counts in central zone		1.43 ± 0.37	3.35 ± 0.83
	Percent rearing counts in central zone		3.47 ± 0.80	3.88 ± 0.44
Elevated plus maze	Percent time in open arm	10 control	49.99 ± 2.97	51.91 ± 3.28
		10 PERM		
	Percent open arm entries		41.97 ± 2.93	40.83 ± 2.04

In the EPM, statistical tests computed on the percent open arm entries (df = 18, t = -0.319, *P* > 0.05) and on the percent time spent in the open arms (df = 18, t = 0.431, *P* > 0.05) revealed no significant group differences, reflecting the same level of anxiety-like responses in PERM-treated rats and in controls.

### Experiment 5. Pain sensitivity in adult rats exposed neonatally to permethrin

Statistical test computed on hot-plate and tail flick latencies in the PERM-treated versus control rats failed to reveal a significant difference at pain sensitivity attributable to PERM treatment made in early life (hot-plate: df = 27, t = -0.916, *P* > 0.05; tail immersion: df = 27, t = 2.003, *P* > 0.05) as shown in Table 1.

### Experiment 6. Synaptic morphology in adult rats exposed neonatally to permethrin

**Figure 4:**
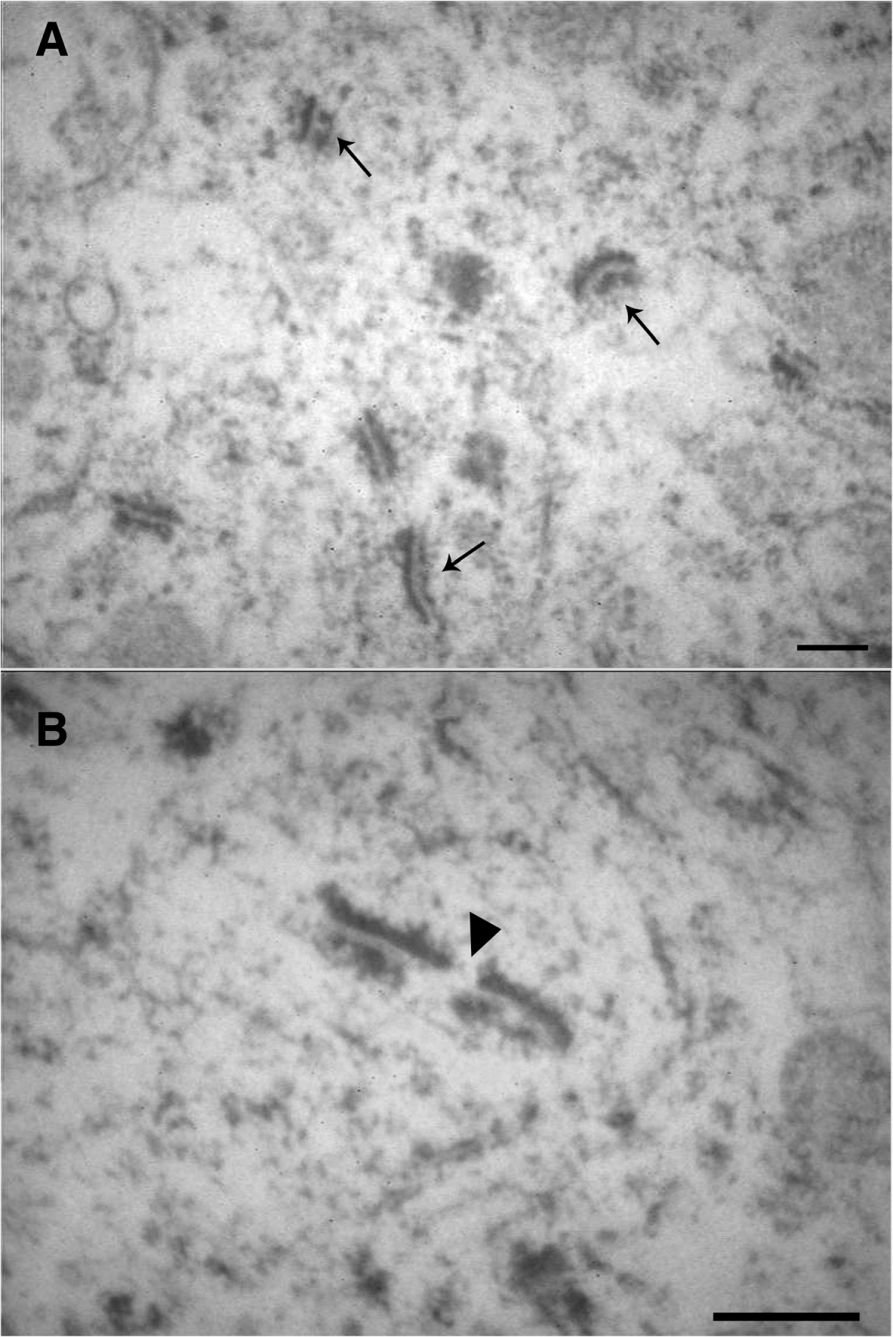
**Electron microscopic picture. A)** synaptic junctions preferentially stained by ethanol-phosphotungstic acid (E-PTA) procedure in the rat hippocampal SMCA1. Pre- and postsynaptic membrane appositions are clearly evidenced as sharp parallel black lines: dotted (arrow) and full line, respectively, against an unstained background. Bar: 0.2 μm; **B)** perforated synapse in rat hippocampal SMCA1 stained by means of E-PTA technique. The evident discontinuity (arrowhead) in the giant contact zone is currently supposed to represent a perforation. Bar: 0.3 μm.

The results are reported in Table 2. Moreover, synaptic junctions and perforated synapses of the rat hippocampal SMCA1 are showed in Figure 4A and B. In SMCA1, PERM-treated rats in comparison to control rats showed a significant decrease of Nv (df = 10, t = 4.597, *P* < 0.001) and Sv (df = 10, t = 4.255, *P* < 0.01), whereas S was not significantly different (df = 10, t = 0.454, *P* > 0.05). In the PERM-treated group, only 2.22% of synapses were identified as perforated, indeed the percentage of perforated synapses seemed to be significantly reduced (df = 10, t = 2.762, *P* < 0.05) in this group compared with control one. In IMLDG, PERM-treated rats in comparison to control group showed a significant decrease of Nv (df = 10, t = 3.220, *P* < 0.01) and Sv (df = 10, t = 4.811, *P* < 0.001), whereas S was not significantly different (df = 10, t = 0.500, *P* > 0.05). In the group treated with PERM, 1.91% of synapses were identified as perforated against 2.64% measured in the control group. Hence, the percentage of perforated synapses is significantly reduced (df = 10, t = 2.584, *P* < 0.05) in PERM treated group compared with control one. In MF, PERM treated rats in comparison to control rats showed a significant decrease of Nv (df = 10, t = 10.250, P < 0.001) and Sv (df = 10, t = 4.963, *P* < 0.001). In the group treated with PERM, S (df = 10, t = -4.246, *P* < 0.01) is significantly increased compared with the control one, whereas the percentage of perforated synapses (df = 10, t = -0.287, *P* > 0.05) was not significantly different.

**Table 2:** The synaptic numeric density, the surface density, the average area (S) and the percentage of perforated synapses (% PS) were calculated in the stratum moleculare of CA1 (SMCA1), mossy fibers (MF) and inner molecular layer of the dentate gyrus (IMLDG) of the hippocampus in control and permethrin (PERM)-treated groups

**Area**	**Group**	**Nv**^**a **^**(N. syn/μm**^**3**^**)**	**Sv**^**b **^**(μm**^**2**^**/μm**^**3**^**)**	**S (μm**^**2**^**)**	**% PS**
**SMCA1**	Control	1.847 ± 0.062	0.112 ± 0.002	0.092 ± 0.007	3.758 ± 0.462
	PERM	1.504 ± 0.039^***^	0.090 ± 0.004^**^	0.088 ± 0.005	2.223 ± 0.308^*^
**MF**	Control	2.431 ± 0.067	0.105 ± 0.003	0.064 ± 0.002	1.393 ± 0.137
	PERM	1.573 ± 0.049^***^	0.081 ± 0.003^***^	0.077 ± 0.002^**^	1.453 ± 0.155
**IMLDG**	Control	2.240 ± 0.085	0.098 ± 0.003	0.065 ± 0.002	2.647 ± 0.195
	PERM	1.949 ± 0.028^**^	0.081 ± 0.001^***^	0.063 ± 0.001	1.911 ± 0.207^*^

Group sizes: control (N = 6), PERM (N = 6). ***P <0.001 versus control group; **P < 0.01 versus control group; *P <0.05 versus control group. ^a^Nv, number of synapses/μm^3^ of tissue; ^b^Sv, overall area of synaptic junctional zones/μm^3^ of tissue.

## Discussion

In previous studies, we demonstrated that rats treated with PERM during the postnatal days 6 to 21 have developed working memory deficits involving the frontal cortico-striatal circuitry later in their lives [11]. Here, in this study, we attempt to elucidate if the hippocampal circuitry is also involved in long-term neurotoxic effects deriving from 15-days exposure to PERM in neonatal age by using hippocampus-dependent tasks never used in our previous studies. Retrieval of memory in aversive conditioning such as contextual fear conditioning, passive avoidance paradigms, and in spatial memory tasks was determined with single probe trials so that the assessment of memory retention did not depend on the ability to relearn across multiple trials.

Behavioral studies have suggested that normal function of hippocampus is necessary for the formation and retrieval of memory in aversive conditioning such as contextual fear conditioning and passive avoidance paradigms, and in spatial memory tasks [27-29].

Several important findings emerged from this study, which focused on behavioral performance of rats in hippocampus-dependent tasks. First of all, PERM treatment in neonatal age altered freezing responses to conditioned fear in adult animals. During the tone-shock conditioning session, the two groups differed in the magnitude of postshock freezing during the ITI periods. In particular, reduced freezing responses were seen in the PERM-treated group versus control group indicating a lower shock reaction. To verify if the results were closely correlated with the loss of sensibility to pain, rats were submitted to nociceptive tests as hot-plate and tail immersion. The data obtained lead us to exclude differences in pain sensibility between PERM-treated rats and controls. However, during the 3-minute baseline period prior to conditioning, locomotor activity in PERM-treated group was higher than that in the control group, suggesting the possibility of locomotor activity effects (Figure 2A). Hence, locomotor behavior in PERM-treated rats and controls was further investigated in the OF. In this task, no differences were observed between groups. These results demonstrate that the locomotor activity of treated rats is increased at the beginning of the test, possibly due to the novelty of the environment, but subsequently disappears as a result of habituation. Thus, since the conditioning session lasts more than 10 min, we exclude the results that were influenced by increased motor activity in the PERM group. Moreover, the treated rats exhibited normal freezing before the conditioning session.

The results concerning contextual and tone conditioning tests showed the treated rats had a decreased freezing to both contextual and tone conditioning. The deficits observed in contextual freezing may be due to hippocampal deficits, whereas amygdala impairment can be responsible for disrupting freezing responses to tone cues [27].

In subsequent experiments, no differences in EPM anxiety-like responses were observed between PERM and control rats. Altogether, these findings rule out the possibility that, in the fear conditioning, the different response between PERM and vehicle-treated rats may depend on differences in an anxiety-like state.

The altered hippocampus functioning in rats treated with PERM in neonatal age was confirmed in the passive avoidance test where the consolidation of the inhibitory avoidance learning, a form of contextual fear memories, appeared to be time-limited. That is, retrieval of inhibitory avoidance was not affected after 24 h long-term recall in a first set of animals, whereas it was impaired 72 h after conditioning in a second set of animals. The data suggested that the hippocampus of PERM-treated rats was working to store memories for a limited period of 24 h. It means that the signaling pathways implicated in the switch from short-term memory, lasting a few minutes, to the long-term memory [30-32] were not impaired after treatment in early life with PERM. Rather these data may suggest that it has limited capacities to retain information after 72 h indicating that the process of memory maintenance in this time frame is compromised. The results, here obtained, allow us to hypothesize that the PERM treatment in early life could compromise, maybe through epigenetic regulations, the structural and molecular processes that normally occur during the stabilization of long-term memories. Data in the literature can support this idea; there are separate mechanisms for initiation of the long-term memory and its stabilization or maintenance. Long-term persistence of memory, but not the initiation, requires the cytoplasmic polyadenylation element-binding proteins (CPEBs) that seem to play an essential role in maintaining the production of synapse-strengthening proteins to stabilize long-term memory [33-35]. By their tendency to form oligomers (more resistant and stable than single monomers) and templates (with a prion-like, self-perpetuating quality) for the formation of new oligomers near the synapses, they could maintain the continuing protein synthesis that stores a memory long after the learning experience has passed.

Furthermore, the data on MWM supported the notion that the spatial reference memories also waned over time in the PERM-treated rats, showing performance impaired on the probe test at 72 h after the end of training, whereas the control group did not show memory impairment. Therefore, the performance of a different set of animals was intact on probe trial after 24 h. In line with the data related to the retrieval of inhibitory avoidance, spatial reference memory was retained no longer than 24 h in PERM-treated rats submitted to a probe test. Similar results were observed by Majumdar *et al*. [36] where they observed that the fruit fly, *Drosophila,* with a mutant form of CPEB protein, lost its ability to form long-term memories 48 h after a memory-forming stimulus, whereas for the first 24 h the memory was not impaired. The authors demonstrated that fruit flies, carrying this mutant form that lacked the normal ability to oligomerize, lost their ability to form long-term memories.

Structural and molecular changes that contribute to the stable maintenance of long-term memory storage suggest the formation of new synaptic connections among neurons that appear as morphological changes such as increases in synaptic spines and synaptic contacts [37,38]. In order to verify that the cognitive impairments in long-lasting memory storage observed in PERM-treated rats were linked to synaptic morphological alterations, we analyzed the density and the size of synapses in various subregions of the hippocampus. The significant decrease of Nv and S_V_ in the SMCA1, MF and IMLDG hippocampal areas together with the decrease of the percent perforated synapses in SMCA1 and IMLDG in PERM-treated rats may explain the impaired synaptic function and subsequent deterioration of spatial and fear memory storage observed 72 h after the end of training in the PERM-treated group. The results were in agreement with other studies that demonstrated impairments in spatial and contextual fear conditioning memories correlated with reduction in hippocampal synapses [39,40]. It is interesting to note that the formation of perforated synapses is an early morphological consequence of synaptic activation characterized by an increased perimeter length and size of the total active synaptic zone that occur transiently in response to enhanced synaptic plasticity [41,42]. According to our data, permethrin exposure could act during the neonatal period to induce irreversible changes in the structural components of synaptic plasticity with consequent detrimental effects on neuroplasticity mechanisms in adult age. Furthermore, alterations of synaptic morphology observed in the three SMCA1, MF and IMLDG regions of permethrin-treated rats disturbs the integrity of the tri-synaptic pathway that is indispensable for contextual learning and spatial memory recall. As recent studies have demonstrated, the hippocampal SMCA1 and IMLDG regions seem to be critically involved in the retrieval of contextual fear conditioning [43,44] and in the memory recall of the MWM task [45-47].

## Conclusions

The present findings support the hypothesis that an environmental insult, such as exposure to PERM pesticide in the postnatal period (from PND6 and PND21) when the rat brain is in a highly active neurodevelopmental period as synaptogenesis, has detrimental effects on hippocampal function and morphology later in adulthood.

Further investigations are in progress to elucidate which signaling pathways involved in the stable maintenance of long-term memory storage are compromised after PERM exposure in early life and the mechanism that is responsible for that.

## Abbreviations

CS: conditioned stimulus; CS-US: conditioned-unconditioned stimuli; EPM: elevated plus maze; IMLDG: inner molecular layer of the dentate gyrus; ITI: intertrial interval; MF: mossy fiber; MWM: Morris water maze; NOAEL: no-observed-adverse-effect level; Nv: number of synapses/μm^3^ of tissue; OF: open field; PERM: permethrin; PND: postnatal day; Ps: percentage of perforated synapses; S: average area of synapse; SMCA1: stratum moleculare of CA1; Sv: overall area of synaptic junctional zones/μm^3^ of tissue; VGSC: voltage-gated sodium channel.

## Competing interests

The authors declare that they have no competing interests.

## Authors’ contributions

M C, D F and RG discussed and commented on the manuscript; PF conducted the experiments on morphology of synapses; RC revised critically the manuscript; MU gave contributions to analysis and interpretation of data; CN contributed to the study design, behavior experiments, analysis of data and manuscript preparation. All authors read and approved the final manuscript.

## References

[B1] U.S. Environmental Protection Agency Proposed Common Mechanism Grouping for the pyrethrins and synthetic pyrethroids, draft http://www.regulations.gov/search/Regs/home.html#documentDetail?R=09000064809a62df

[B2] Barr DB Olsson AO Wong LY Udunka S Baker SE Whitehead RD Magsumbol MS Williams BL Needham LL Urinary concentrations of metabolites of pyrethroid insecticides in the general U.S. population: National Health and Nutrition Examination Survey 1999-2002 Environ Health Perspect J Am Coll Nutr 2010 118 742 748 10.1289/ehp.0901275 PMC2898848 20129874

[B3] Heudorf U Angerer J Metabolites of pyrethroid insecticides in urine specimens: current exposure in an urban population in Germany Environ Health Perspect 2001 109 213 217 10.1289/ehp.01109213 11333180 PMC1240237

[B4] Becker K Seiwert M Angerer J Kolossa-Gehring M Hoppe HW Ball M GerES IV pilot study: assessment of the exposure of German children to organophosphorus and pyrethroid pesticides Int J Hyg Environ Health 2006 209 221 233 10.1016/j.ijheh.2005.12.002 16461005

[B5] Naeher LP Tulve NS Egeghy PP Barr DB Adetona O Fortmann RC Needham LL Bozeman E Hilliard A Sheldon LS Organophosphorus and pyrethroid insecticide urinary metabolite concentrations in young children living in a southeastern United States city Sci Total Environ 2010 408 1145 1153 10.1016/j.scitotenv.2009.10.022 19896164

[B6] Cantalamessa F Acute toxicity of two pyrethyroids, permethrin, and cypermethrin in neonatal and adult rats Arch Toxicol 1993 67 510 513 10.1007/BF01969923 8240001

[B7] Meacham CA Brodfuehrer PD Watkins JA Shafer TJ Developmentally-regulated sodium channel subunits are differentially sensitive to alpha-cyano containing pyrethroids Toxicol Appl Pharmacol 2008 231 273 281 10.1016/j.taap.2008.04.017 18538810

[B8] U.S. Environmental Protection Agency Office of Prevention Pesticide and Toxic Substances: Permethrin Facts http://www.epa.gov/oppsrrd1/REDs/permethrin_red.pdf

[B9] Imamura L Hasegawa H Kurashina K Matsuno T Tsuda M Neonatal exposure of newborn mice to pyrethroid (permethrin) represses activity-dependent c-fos mRNA expression in cerebellum Arch Toxicol 2002 76 392 397 10.1007/s00204-002-0358-2 12111003

[B10] Nasuti C Gabbianelli R Falcioni ML Di Stefano A Sozio P Cantalamessa F Dopaminergic system modulation, behavioral changes, and oxidative stress after neonatal administration of pyrethroids Toxicology 2007 18 194 205 17140720 10.1016/j.tox.2006.10.015

[B11] Nasuti C Carloni M Fedeli D Gabbianelli R Di Stefano A Cerasa LS Silva I Domingues V Ciccocioppo R Effects of early life permethrin exposure on spatial working memory and on monoamine levels in different brain areas of pre-senescent rats Toxicology 2013 303 162 168 23174539 10.1016/j.tox.2012.09.016

[B12] Carloni M Nasuti C Fedeli D Montani M Amici A Vadhana MS Gabbianelli R The impact of early life permethrin exposure on development of neurodegeneration in adulthood Exp Gerontol 2012 47 60 66 10.1016/j.exger.2011.10.006 22056222

[B13] Carloni M Nasuti C Fedeli D Montani M Vadhana MS Amici A Gabbianelli R Early life permethrin exposure induces long-term brain changes in Nurr1, NF-kB and Nrf-2 Brain Res 2013 1515 19 28 23566817 10.1016/j.brainres.2013.03.048

[B14] Vadhana MS Carloni M Nasuti C Fedeli D Gabbianelli R Early life permethrin insecticide treatment leads to heart damage in adult rats Exp Gerontol 2011 46 731 738 10.1016/j.exger.2011.05.005 21616133

[B15] Vadhana MS Siva Arumugam S Carloni M Nasuti C Gabbianelli R Early life permethrin treatment leads to long-term cardiotoxicity Chemosphere 2013 93 1029 34 10.1016/j.chemosphere.2013.05.073 23806482

[B16] Romijn HJ Hofman MA Gramsbergen A At what age is the developing cerebral cortex of the rat comparable to that of the full-term newborn human baby? Early Hum Dev 1991 26 61 67 10.1016/0378-3782(91)90044-4 1914989

[B17] Clancy B Finlay BL Darlington RB Anand KJ Extrapolating brain development from experimental species to humans Neurotoxicology 2007 28 931 937 10.1016/j.neuro.2007.01.014 17368774 PMC2077812

[B18] Vaiserman AM Carstensen B Voitenko VP Tronko MD Kravchenko VI Khalangot MD Mechova LV Seasonality of birth in children and young adults (0-29 years) with type 1 diabetes in Ukraine Diabetologia 2007 50 32 35 17093948 10.1007/s00125-006-0456-4

[B19] Barker DJ The developmental origins of adult disease J Am Coll Nutr 2004 23 588S 595S 10.1080/07315724.2004.10719428 15640511

[B20] Goosens KA Maren S Contextual and auditory fear conditioning are mediated by the lateral, basal, and central amygdaloid nuclei in rats Learn Mem 2001 8 148 155 10.1101/lm.37601 11390634 PMC311374

[B21] Zacher C Gass P Unsicker K Von Bohlen Und Halbach O Age-related alterations in hippocampal spines and deficiencies in spatial memory in mice J Neurosci Res 2006 83 525 531 10.1002/jnr.20759 16447268

[B22] Selkoe DJ Alzheimer’s disease is a synaptic failure Science 2002 298 789 791 10.1126/science.1074069 12399581

[B23] Turner R Anti-inflammatory agent Screening Methods in Pharmacology 1965 New York, London: Academic Press 12 18

[B24] Janssen PAJ Niemegeers CJE Dony JGH The inhibitory effect of fentanyl and other morphine-like analgesics in the warm water-induced tail withdrawal reflex in rats Arzneim-Forsch Drug Res 1963 6 502 507 13957426

[B25] Platano D Fattoretti P Balietti M Giorgetti B Casoli T Di Stefano G Bertoni-Freddari C Aicardi G Synaptic remodeling in hippocampal CA1 region of aged rats correlates with better memory performance in passive avoidance test Rejuv Res 2008 11 341 348 10.1089/rej.2008.0725 18442322

[B26] Coggeshall RE Lekan HA Methods for determining numbers of cells and synapses: a case for more uniform standards of review J Comp Neurol 1996 364 6 15 10.1002/(SICI)1096-9861(19960101)364:1<6::AID-CNE2>3.0.CO;2-9 8789272

[B27] Phillips RG LeDoux JE Differential contribution of amygdala and hippocampus to cued and contextual fear conditioning Behav Neurosci 1992 106 274 285 1590953 10.1037//0735-7044.106.2.274

[B28] Ambrogi Lorenzini CG Baldi E Bucherelli C Sacchetti B Tassoni G Role of the ventral hippocampus in acquisition, consolidation and retrieval of rat’s passive avoidance response memory trace Brain Research 1997 768 242 248 10.1016/S0006-8993(97)00651-3 9369321

[B29] Morris RGM Garrud P Rawlins JNP O’Keefe J Place navigation impaired in rats with hippocampal lesions Nature 1982 297 681 683 10.1038/297681a0 7088155

[B30] Kandel ER The molecular biology of memory storage: a dialogue between genes and synapses Science 2001 294 1030 1038 10.1126/science.1067020 11691980

[B31] Yin JC Del Vecchio M Zhou H Tully T CREB as a memory modulator: induced expression of a dCREB2 activator isoform enhances long-term memory in Drosophila Cell 1995 81 107 115 10.1016/0092-8674(95)90375-5 7720066

[B32] Martin KC Kandel ER Cell adhesion molecules, CREB, and the formation of new synaptic connections Neuron 1996 17 567 570 10.1016/S0896-6273(00)80188-9 8893013

[B33] Theis M Si K Kandel ER Two previously undescribed members of the mouse CPEB family of genes and their inducible expression in the principal cell layers of the hippocampus Proc Natl Acad Sci U S A 2003 100 9602 9607 10.1073/pnas.1133424100 12871996 PMC170964

[B34] Pavlopoulos E Trifilieff P Chevaleyre V Fioriti L Zairis S Pagano A Malleret G Kandel ER Neuralized1 activates CPEB3: a function for nonproteolytic ubiquitin in synaptic plasticity and memory storage Cell 2011 147 1369 1383 10.1016/j.cell.2011.09.056 22153079 PMC3442370

[B35] Udagawa T Swanger SA Takeuchi K Kim JH Nalavadi V Shin J Lorenz LJ Zukin RS Bassell GJ Richter JD Bidirectional control of mRNA translation and synaptic plasticity by the cytoplasmic polyadenylation complex Mol Cell 2012 47 253 266 10.1016/j.molcel.2012.05.016 22727665 PMC3408552

[B36] Majumdar A Cesario WC White-Grindley E Jiang H Ren F Khan MR Li L Choi EM Kannan K Guo F Unruh J Slaughter B Si K Critical role of amyloid-like oligomers of Drosophila Orb2 in the persistence of memory Cell 2012 148 515 529 10.1016/j.cell.2012.01.004 22284910

[B37] Fischer A Sananbenesi F Pang PT Lu B Tsai LH Opposing roles of transient and prolonged expression of p25 in synaptic plasticity and hippocampus-dependent memory Neuron 2005 48 825 838 10.1016/j.neuron.2005.10.033 16337919

[B38] Guan JS Haggarty SJ Giacometti E Dannenberg JH Joseph N Gao J Nieland TJ Zhou Y Wang X Mazitschek R Bradner JE DePinho RA Jaenisch R Tsai LH HDAC2 negatively regulates memory formation and synaptic plasticity Nature 2009 459 55 60 10.1038/nature07925 19424149 PMC3498958

[B39] Saxe MD Battaglia F Wang JW Malleret G David DJ Monckton JE Garcia AD Sofroniew MV Kandel ER Santarelli L Hen R Drew MR Ablation of hippocampal neurogenesis impairs contextual fear conditioning and synaptic plasticity in the dentate gyrus Proc Natl Acad Sci U S A 2006 103 17501 17506 10.1073/pnas.0607207103 17088541 PMC1859958

[B40] Nicholson DA Yoshida R Berry RW Gallagher M Geinisman Y Reduction in size of perforated postsynaptic densities in hippocampal axospinous synapses and age-related spatial learning impairments J Neurosci 2004 24 7648 7653 10.1523/JNEUROSCI.1725-04.2004 15342731 PMC6729620

[B41] Geinisman Y Perforated axospinous synapses with multiple, completely partitioned transmission zones: probable structural intermediates in synaptic plasticity Hippocampus 1993 3 417 433 10.1002/hipo.450030404 8269034

[B42] Sorra KE Fiala JC Harris KM Critical assessment of the involvement of perforations, spinules, and spine branching in hippocampal synapse formation J Comp Neurol 1998 24 225 240 9700568

[B43] Goshen I Brodsky M Prakash R Wallace J Gradinaru V Ramakrishnan C Deisseroth K Dynamics of retrieval strategies for remote memories Cell 2011 147 678 689 10.1016/j.cell.2011.09.033 22019004

[B44] Liu X Ramirez S Pang PT Puryear CB Govindarajan A Deisseroth K Tonegawa S Optogenetic stimulation of a hippocampal engram activates fear memory recall Nature 2012 484 381 385 22441246 10.1038/nature11028 PMC3331914

[B45] Nakazawa K Quirk MC Chitwood RA Watanabe M Yeckel MF Sun LD Kato A Carr CA Johnston D Wilson MA Tonegawa S Requirement for hippocampal CA3 NMDA receptors in associative memory recall Science 2002 297 211 218 10.1126/science.1071795 12040087 PMC2877140

[B46] Zelikowsky M Bissiere S Fanselow MS Contextual fear memories formed in the absence of the dorsal hippocampus decay across time J Neurosci 2012 32 3393 3397 10.1523/JNEUROSCI.4339-11.2012 22399761 PMC3306617

[B47] Montgomery SM Buzsaki G Gamma oscillations dynamically couple hippocampal CA3 and CA1 regions during memory task performance PNAS 2007 107 14495 14500 17726109 10.1073/pnas.0701826104 PMC1964875

